# Numerical Encodings of Amino Acids in Multivariate Gaussian Modeling of Protein Multiple Sequence Alignments

**DOI:** 10.3390/molecules24010104

**Published:** 2018-12-28

**Authors:** Patrice Koehl, Henri Orland, Marc Delarue

**Affiliations:** 1 Department of Computer Science, University of California, Davis, CA 95211, USA; 2 Institut de Physique Théorique, CEA Saclay, 91191 Gif-sur-Yvette CEDEX, France; henri.orland@cea.fr; 3 Department of Structural Biology and Chemistry and UMR 3528 du CNRS, Institut Pasteur, 75015 Paris, France; delarue@pasteur.fr

**Keywords:** multiple sequence alignment, covariation, contact predictions

## Abstract

Residues in proteins that are in close spatial proximity are more prone to covariate as their interactions are likely to be preserved due to structural and evolutionary constraints. If we can detect and quantify such covariation, physical contacts may then be predicted in the structure of a protein solely from the sequences that decorate it. To carry out such predictions, and following the work of others, we have implemented a multivariate Gaussian model to analyze correlation in multiple sequence alignments. We have explored and tested several numerical encodings of amino acids within this model. We have shown that 1D encodings based on amino acid biochemical and biophysical properties, as well as higher dimensional encodings computed from the principal components of experimentally derived mutation/substitution matrices, do not perform as well as a simple twenty dimensional encoding with each amino acid represented with a vector of one along its own dimension and zero elsewhere. The optimum obtained from representations based on substitution matrices is reached by using 10 to 12 principal components; the corresponding performance is less than the performance obtained with the 20-dimensional binary encoding. We highlight also the importance of the prior when constructing the multivariate Gaussian model of a multiple sequence alignment.

## 1. Introduction

The current understanding of the relation between the sequence of a protein and its structure remains limited. As of November 2018, we know a lot about protein sequences (more than hundred twenty millions of them, if one refers to RefSeq, the NCBI Reference Sequence Database, https://www.ncbi.nlm.nih.gov/refseq/, [1]), more and more about their structures (there are more than 130,000 structures of proteins in the Protein Data Bank, PDB, http://www.rcsb.org, [2]), but we still have difficulties in deciphering the rules that relate a sequence to its corresponding three-dimensional structure, a challenge usually referred to as the “holy grail” of computational structural molecular biology [3]. In this paper, we focus on one of those rules, namely the prediction of geometric proximity of residues (also called residue contacts) in a protein structure from the variability of the amino acid sequence of that protein within its family.

The protein sequences of the same protein from different species vary much more than the three-dimensional structure they all adopt. For residues in such a protein to remain in close proximity, one hypothesis that has been put forward is that the corresponding positions in the protein sequences experience correlated mutations throughout evolution: In the event that one residue of such a pair mutates, the effect of this mutation is likely to be accommodated by a corresponding mutation of the other residue of this pair. This hypothesis, however, might not be true, as discussed in detail by Talavera et al. [4]. In their studies, they have shown that coordinated substitutions require unfeasibly long times. They do find that pairs of residues with strong covariation signals tend to have low evolutionary rates, with such residues being located primarily in the cores of proteins, thereby in close proximity. It remains that, in both scenarios, namely co-variations due to molecular co-evolution or due to low evolutionary rates within the protein core, if we can detect co-variation between positions within a multiple sequence alignment (MSA), we should be able to predict geometric contacts between residues in the corresponding protein structure. We should note also that co-variations do not always mean spatial proximity; this was discussed in length in the recent analysis of coevolution between distant residues by Baker and co-workers [5].

The possibility to detect co-variations within a MSA has significantly increased with the surge in the number of protein sequences that are available. The related search for an inference between those co-variations and actual contacts in protein has been the focus of decades of research, and recent breakthroughs have lead to increased precision in predicting contacts from sequence alone (for recent reviews on this topic, see [6,7,8,9]). It should be noted that analyses of covariation have been used with success outside the protein structure prediction problem, from the prediction of the structure of protein complexes [8,10], and protein conformational transitions [11,12], RNA structure prediction [13,14,15], and prediction of ordered states for disordered proteins [16] to the prediction of mutation effects in proteins [17].

The idea of inferring spatial proximity from direct covariance of amino acid distributions in pairs of position within a MSA originated from the early 1980s as it was used then for predicting virus functions [18] and a little later (in the 1990s) to predict contacts in proteins [19,20]. Success, however, was limited, possibly for a reason that can be characterized as follows [7,21]: When a residue *i* is in close proximity to a residue *j*, and *j* itself is in contact with another residue *k*, then *i* and *k* are likely to exhibit covariation, even if they are not close in space. Prediction of spatial proximity from co-variations therefore require that direct interactions (between *i* and *j*, and between *j* and *k*) be distinguished from possible indirect interactions (between *i* and *k*). Two approaches are currently being developed for this task: Those that generate a statistical model for protein sequences by using techniques from statistics or physics and those that learn this model directly from the data by using machine-learning techniques. Among the statistical techniques used for analyzing co-variations in MSA, we can cite sequence-based probabilistic formalisms, proposed as early as 2002 [22], message-passing algorithms [23], mean-field methods [21], and pseudo-likelihood [24,25] or multivariate Gaussian [26,27] approximations. Here we focus on the latter, namely a representation of covariations using a multivariate Gaussian model.

The multivariate Gaussian modeling of a MSA is an approximation. It relies on a Gaussian interaction model in which amino acids are represented by small real-valued vectors. The covariance matrix of this Gaussian model is estimated from the observed covariance matrix computed from the MSA, and its inverse, namely the precision or concentration matrix, is used to predict the direct contacts in the corresponding protein structure [26]. In its current implementations, namely PSICOV [26], and GaussDCA [27], the vector r(a) representing an amino acid of type *a* is defined by a binary encoding, with r(i)=1 if i=a, and zero otherwise. This is a vector of size 20, to account for the 20 types of amino acids. A sequence *S* in an MSA of length *N* is then represented with an array of numerical values of size 20N, and the corresponding observed covariance matrix for the MSA is of size 20N×20N. The large size of this matrix leads to a problem related to under-sampling: Not every amino-acid will be observed at every site of the MSA, leading to a covariance matrix that is singular. To compute the inverse of this singular covariance matrix, Jones et al. proposed a constrained sparse inverse estimation using the graphical Lasso method [26], while Baldassi et al. added a prior distribution to remove the singularity, much akin to the concept of pseudo counts [27]. In this paper, we propose complementary approaches to reduce the impact of under-sampling, in which the size of the vector representing an amino acid is reduced and numerical components are introduced.

When writing down a protein sequence, amino acids are usually pictured either with a one-letter code, or a three-letter code. Both encodings are simply representations of the amino acid name, and therefore do not contain any information per se. Alternately, encoding amino acids as vectors of numerical values has the advantage of increasing the information content of a protein sequence, should those numerical values represent physico-chemical, or other properties of the amino acids. Such representations are expected to allow finer analyses of the functions of the proteins they represent. French and Robson [28], Swanson [29], and Kidera et al. [30] may have been the first to implement this concept in the early 1980s. Swanson observed that the 20 × 20 Dayhoff substitution matrix [31] is akin to mapping amino acids into a 20-dimensional feature space. By applying dimension reduction techniques, three lower dimensional representations of amino acids were proposed in 1D, 2D, and 3D spaces, with the 2D version expected to be the most reasonable as it was consistent with other amino acid properties [29]. In parallel, Kidera et al. proposed to encode each amino acid with ten independent factors obtained by principal component analyses (PCA) of more than one hundred and eighty properties of the twenty amino acids [30]. Such a representation has been used to analyze protein sequences using Fourier analysis [32,33,34], with applications to fold recognition for homology modeling [35]. It should be noted also that a numerical encoding of amino acids enables the definition of sophisticated metrics for comparing sequences [36]. It also leads to the concept of geometric representations of protein sequences, and their applications for sequence classification and protein fold recognition (see [37] and references therein).

This paper draws from this concept and describes a feature-based representation of protein sequences. In this representation, each amino acid is encoded by a unique vector of features that are derived either from the physico-chemical properties of the amino acid considered or from a lower dimensional representation of an amino substitution matrix. We analyze how those reduced representations compare to the binary encoding currently used in multivariate Gaussian models for residue contact prediction, with respect to the need for correction for under-sampling, as well as with respect to their prediction accuracy.

The paper is organized as follows. The following section covers the concept of a multidimensional Gaussian model of an MSA and its application to contact prediction. The result section describes applications of different encoding of amino acids for protein contact prediction, using a test set of 150 multiple sequence alignments originally developed as a test set for PSICOV [26]. We then conclude with a discussion of future research directions.

## 2. A Gaussian Model for Protein Contact Prediction

The multivariate Gaussian model is well-studied in statistics; it has been introduced independently for analyzing multiple sequence alignments by Jones et al. [26] and Baldassi et al. [27]. Here we briefly review the main ideas behind this model with respect to its application for contact prediction in proteins from sequence information contained in MSAs.

### 2.1. A Multiple Sequence Alignment and Its Numeric Representation

The input data is the MSA generated from multiple homologous sequences for the same protein. Such a MSA is described by *N* aligned protein sequences of length *L*. Each sequence $S_{n}$ in the alignment is a string of characters taken from an alphabet of size 21 including the 20 standard amino acids plus one character for gaps. An amino acid of type *i* is then represented by a vector $r_{i}$ whose size *s* and component values depend on the encoding that is chosen. In the work of Jones et al. [26] and Baldassi et al. [27], *s* is set to 20, and $r_{i}\left(k\right)=\delta_{ik}$ where δ is the Kronecker delta. Namely, $r_{i}$ is a vector whose components are 0, except for the component index corresponds to the type *i*, in which case it is 1. The vector representing a gap is always set to 0. In this study, we will explore different encodings for $r_{i}$.

Once the encoding is defined, the letter-based sequence $S_{n}$ of length *L* is represented by a real-valued sequence $X_{n}$, of length Ls, obtained by replacing each letter by its corresponding vector in the encoding.

The empirical mean sequence over the MSA is then computed as:

(1) $$\bar{X}=\frac{1}{N}\sum_{n=1}^{N}X_{n}$$

Similarly, the empirical covariance matrix *C* for the MSA is computed as:

(2) $$\bar{C}=C\left(MSA,\bar{X}\right)=\frac{1}{N}\sum_{n=1}^{N}{\left(X_{n}-\bar{X}\right)}^{T}\left(X_{n}-\bar{X}\right)$$

*C* is a matrix of size Ls×Ls.

### 2.2. A Gaussian Model for the Alignment

The main assumption in this approach is that each sequence $X_{n}$ in the MSA is drawn from a multivariate Gaussian distribution characterized by a mean vector μ and a covariance matrix ∑, with the probability:

(3) $$P\left(X_{n}|\mu ,\sum \right)={\left(2\pi \right)}^{-\frac{Ls}{2}}{\left|\sum \right|}^{-\frac{1}{2}}\exp\left[-\frac{1}{2}{\left(X_{n}-\mu \right)}^{T}\sum^{-1}\left(X_{n}-\mu \right)\right]$$

in which |∑| is the determinant of ∑.

Assuming that the *N* sequences in the MSA are statistical independent, the probability, or likelihood of the data under this model is given by

(4) $$\begin{array}{cc} P(MSA|\mu ,\sum ) & =\prod_{n=1}^{N}P\left(X_{n}|\mu ,\sum \right)\\ & ={\left(2\pi \right)}^{-\frac{NLs}{2}}{\left|\sum \right|}^{-\frac{N}{2}}\exp\left[-\frac{N}{2}tr\left(\sum^{-1}C\left(MSA,\mu \right)\right)\right] \end{array}$$

with C(MSA,μ) the empirical covariance matrix given by Equation (2), but computed with the (unknown) true mean of the population of sequences, μ, instead of the empirical mean $\bar{X}$. Using the maximum likelihood estimator for P(MSA|μ,∑), the mean vector and the covariance matrix of the multivariate model can be estimated as $\mu =\bar{X}$ and $\sum =\bar{C}=C\left(MSA,\bar{X}\right)$, where the over line highlights that these are empirically-derived values [38].

We note that the independence of the sequences in the alignment is a strong assumption that is usually not satisfied in a MSA, due to phylogenic relationships between the sequences. To alleviate this problem, a weight is usually associated with each sequence $S_{n}$ [25,27]. We use the definitions of the weights proposed by Baldassi et al. [27], see Material and Methods below for how they are computed.

### 2.3. Extracting Coupling Information from the Parameters of the Gaussian Model

In the mean-field approximation, there is a simple relationship between the coupling matrix *J* and the empirical covariance matrix *C*, namely that $J=-{\bar{C}}^{-1}$. A similar relationship has been used under the Gaussian model. We can see why the inverse is used instead of the direct covariance from two perspectives. Notice first that the term in the exponential of the Gaussian model (Equation (3)) can be rewritten as:

(5) $${\left(X_{n}-\mu \right)}^{T}\sum^{-1}\left(X_{n}-\mu \right)=\sum_{i=1}^{N}\sum_{j=1}^{N}\left(X_{i}-\mu_{i}\right)\left(\sum^{-1}\right)\left(i,j\right)\left(X_{j}-\mu_{j}\right)$$

This shows that $\left(\sum^{-1}\right)\left(i,j\right)$ serves as a coupling between positions *i* and *j* in the MSA. From a more statistical point of view, $\sum^{-1}$ is the precision, also called concentration, matrix of the Gaussian model. The precision matrix captures conditional dependencies, which is expected to help differentiate direct from indirect couplings that cannot be achieved from the covariance matrix directly.

Computation of $J={\bar{C}}^{-1}$ however assumes that the matrix *C* is full rank. This is unlikely with a 20 letter alphabet, as not every amino acid will be observed at every site of the MSA, even in very large protein families. There are many methods to alleviate this problem, from the computation of a pseudo inverse, the estimation of a sparse inverse using Lasso methods [26], to the addition of a prior distribution. In this work, we use the latter, following the original idea of Baldassi et al. [27]. Briefly, we generate a prior as follows. If $r_{i}$ is the vector representing an amino acid of type *i*, we generate the mean vector $\bar{r}$ and covariance Cr for a distribution sampled uniformly over the 20 types of amino acids,

(6) $$\begin{array}{ccc} \bar{r} & = & \frac{1}{20}\sum_{i=1}^{20}r_{i}\\ Cr & = & \frac{1}{20}\sum_{i=1}^{20}{\left(r_{i}-\bar{r}\right)}^{T}\left(r_{i}-\bar{r}\right) \end{array}$$

The prior $P(\mu_{P},C_{P})$ for the whole MSA is then defined by setting its mean $\mu_{P}={\left[\bar{r}\ldots \bar{r}\right]}^{T}$ (with *L* copies of the mean $\bar{r}$), and its covariance $C_{P}$ such that $C_{P}\left(i,i\right)=Cr$ for any position *i* in the MSA, and $C_{P}\left(i,j\right)=0$ for any pairs of position *i* and *j* in the MSA. This prior is specific to the MSA, and derived from a uniform distribution of amino acids at each position in the MSA. The corrected covariance $C_{M}$ for the MSA is then defined as:

(7) $$C_{M}=\lambda C_{p}+\left(1-\lambda \right)\bar{C}+\lambda \left(1-\lambda \right){\left(\bar{X}-\mu_{P}\right)}^{T}\left(\bar{X}-\mu_{P}\right)$$

where $\bar{X}$ and $\bar{C}$ are the empirical mean sequence and covariance of the MSA, as defined by Equations (1) and (2), respectively, and λ is a parameter that controls the weight given to the prior.

The coupling matrix *J* is computed from the corrected covariance matrix by matrix inversion. It should be noted that *J* is a matrix of size Ls×Ls, i.e., that the “coupling” between a position *i* and *j* in the MSA is given by a s×s matrix. Each of these small matrices are then transformed into an actual score $SC_{ij}$, using the Frobenius norm of J(i,j), and the matrix SC is then corrected by applying an average-product-correction, (APC) [25,39]. Details on those two steps are provided in the Material and Methods section.

## 3. Results

We have implemented the multivariate Gaussian model described above in a program called GaussCovar, following the algorithm described in the Material and Methods section. GaussCovar mimics the program GaussDCA proposed by Baldassi et al. [27], with the significant difference that we explore different vector representations of the amino acids, when converting a multiple sequence alignment into a numerical matrix from which contacts can be predicted. In this section, we describe and apply some of those representations, from the binarized 20-dimensional vectors originally used by Baldassi et al. [27] and by Jones et al. [26], a single value for each amino acid, derived from the AAindex database [40,41], to vectors derived from projections of BLOSUM matrices onto their principal components. We are particularly interested in assessing the balance between performance, and the need to add prior information to the Gaussian model.

GaussCovar takes as input a multiple sequence alignment and outputs a list of predicted contacts, in decreasing order of scores. Just like GaussDCA, GaussCovar is fast: Its most computer intensive parts are the computations of the sequence weights, of order $N^{2}$ where *N* is the number of sequence in the MSA, and the computation of the inverse of the covariance matrix, of order $L^{3}$ where *L* is the length of the sequences in the MSA.

We have tested GaussCovar with different amino acid encodings on two different datasets, each with 150 families. We used two different datasets to assess whether the results we obtain are consistent over those datasets, in which case they are likely indicators of the properties of GaussCovar and of the amino acid encodings, or different on those datasets, in which case they are more likely to be indicators of confounding effects specific to the construction of the datasets. The first dataset, PSICOV, was originally used for assessing the performance of the namesake program PSICOV [26]. The MSAs in PSICOV have a number of sequences *N* between 511 and 74836, and a length *L* between 50 and 266. In parallel, we have used NOUMENON, a more recent dataset based also on 150 protein families, significantly different however in its conception than PSICOV [42]. NOUMENON is designed to be as bias-free as possible, by only considering protein sequences whose homologues in the MSAs have little, or no structural information available in the PDB, the database of protein structures. As such, NOUMENON is expected to mimic more realistic applications of residue contact predictions. NOUMENON contains MSAs with a more diverse number of sequences than PSICOV, ranging from N=2 to N=513,407, but with similar lengths *L*, between 64 and 275. Note that the actual number of sequences may be misleading and that a better measure of the information content of a MSA is its effective number of sequences, $M_{eff}$. This will be discussed below. PSICOV and NOUMENON are described in more details in the Material and Methods section below, with links to where their authors have made them available.

Results are presented as averages over the 150 protein families for each dataset, unless specified. In particular, we generate the precision or positive predictive value (PPV) curves as functions of the number of predicted contacts considered. More specifically, PP(k) is the rate of true positive contacts among the top *k* predicted contacts, where “true” refers to a contact that exists in a gold standard protein structure, i.e., a contact between two residues that are close in space (see Material and Methods for details). As an overall measure of quality, we compute the area under the PPV curve from k=1 to k=200, referred to as AUC200. Note that the higher the AUC200, the better the prediction. The best possible value for AUC200 is 200.

### 3.1. Amino Acid Representation 1: 20-Dimensional Binarized Vectors

**Figure 1:**
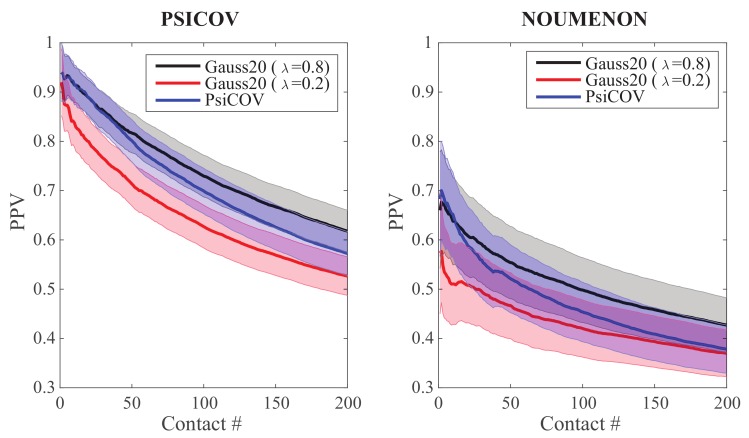
Precision Positive Values (PPV) plotted against the number of predicted contacts for the PSICOV dataset (**left**) and the NOUMENON dataset (**right**). PPV(k) is the rate of correctly predicted contacts within the first *k* contacts. Data for the Gaussian models were generated with GaussCovar, with a 20-dimensional binarized vector representing amino acids, with λ=0.8 (black curve), or λ=0.2 (red curve). Data for the PSICOV predictions (blue curve) on the PSICOV dataset were obtained from the data provided by the authors [26], while the similar data on the NOUMENON dataset were generated by running directly PSICOV (see text for details). All PPV curves are arithmetic means over 150 protein families. The shaded areas represent standard deviations.

As a first test, we ran GaussCovar on the two datasets of 150 protein families presented above using a simple twenty dimensional (20D) representation of amino acids. To emphasize that we have used a 20D representation, we refer to these experiments as Gauss20. In this representation, an amino acid of type *i* is represented with a vector of 20 zeros, except at position *i* where it is given the value 1. In Figure 1, we compare the performances of Gauss20 for two different values of λ (the weight of the prior, see Equation (7)), namely λ=0.8 and λ=0.2, against the performance of the PSICOV program, which uses the same representation of amino acids. Results for PSICOV on its namesake dataset were kindly provided by its authors [26], along with the dataset itself. In parallel, we obtained the program PSICOV (http://bioinfadmin.cs.ucl.ac.uk/downloads/PSICOV/), and ran it over all 150 MSAs in the NOUMENON dataset. Following the advices provided in the original PSICOV paper [26], we set the lasso regularization parameter ρ to a constant value of 0.001 for all MSAs with more than 100 sequences. We found that PSICOV struggles to converge on a solution for MSAs with a smaller number of sequences; for all of those, we increased the ρ parameter to 0.01.

We observe similar levels of performance between PSICOV and Gauss20 with λ=0.8 (the two-tailed t-probabilities computed when comparing the distributions of Gauss20 (λ=0.8) and PSICOV results over the 150 families are always greater than 0.05 at all levels of contacts considered), and significantly lower performance for the latter when λ is set to 0.2 (where “significance” comes from the fact that the two-tailed t-probabilities computed when comparing the distributions of Gauss20 (λ=0.8) and Gauss20 (λ=0.8) results over the 150 families are smaller than 0.05 at all levels of contacts considered, with the exception of the first few data points, when the number of contacts is below 5). Similar relative differences between the performances of Gauss20 (λ=0.8) and Gauss20 (λ=0.2), and between the performances of Gauss20 (λ=0.8) and PSICOV are observed between the two datasets considered. Of interest, however, we note that their absolute performances vary greatly from one dataset to the other, with all three methods performing better on the PSICOV dataset, with approximately a 50% performance drop on the NOUMENON dataset. This behavior was already reported [42] for the PSICOV program on the same datasets, and for the program CCMpred that implements a method derived from statistical mechanics [43]. The drop is assumed to be related to over-estimation of the performances of contact prediction methods on the PSICOV dataset due to bias in the selection of the protein families it contains, a bias that was presumably removed when designing the NOUMENON dataset.

**Figure 2:**
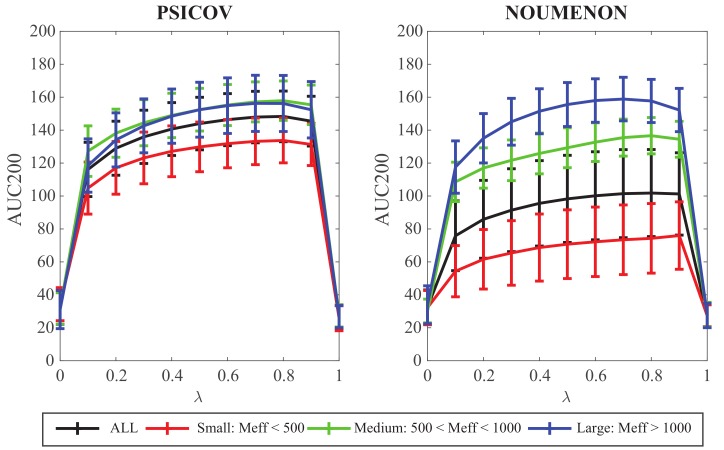
AUC200 plotted against λ, the weight of the prior in the Gaussian model for the PSICOV dataset (**left**) and the NOUMENON dataset (**right**). AUC200, with values between 0 and 200, is a measure of performance, with higher values indicating better performance. The 150 multiple sequence alignment (MSAs) in the datasets are broken down into 3 groups, small ($M_{eff}<500$, medium ($500\leq M_{eff}<1000$), and large ($1000\leq M_{eff}$).

To further investigate the impact of λ, we explored the whole range of its values between 0 and 1, with a step size of 0.1. Results are shown in Figure 2. The performance of the Gaussian model Gauss20 is clearly improved as λ is increased, with a peak at λ=0.8, for both the PSICOV dataset and the NOUMENON dataset. As above, we see a drop of performance on the latter. The need for a large value for λ was already described for GaussDCA and is therefore not a surprise here as GaussCovar is equivalent to GaussDCA when a 20D binarized representation of amino acids. The need for a high value for λ can be assigned to the large sparsity of the numerical representation of MSAs with this amino acid representation. Even for MSA with a large number of sequences, it is unlikely that all amino acids have been observed at each position of the MSA. It is interesting however that the addition of a very simple prior improves significantly the performance of GaussCovar. This prior by itself has no predictive power, as observed in Figure 2 for λ=1.0.

The 150 protein families included in each of the two datasets considered are quite diverse, within the dataset themselves, and between the datasets. They include alignments with a number of sequences *N* varying from 511 to 74,836 for PSICOV, and between 2 and 513,407 for NOUMENON. We note, however, that *N* is not a good measure of the information content of a multiple sequence alignment: An MSA with a large number of very similar sequences will likely not be more informative (with respect to contact prediction) than a MSA with a smaller number of sequences, but with more diversity. One way to account for this effect is to consider the effective number $M_{eff}$ of sequences of a MSA. There are many ways to define this effective number (see for example [44]). Here we rely on the definition provided by Baldassi et al. [27]; we briefly explain how it is computed in the Material and Methods section. To assess whether the need to add the prior differs for alignments with different information contents, we repeated the analyses presented above on three subsets of the families, namely those with $M_{eff}<500$ (small MSAs), with $500<M_{eff}<1000$ (medium MSAs), and with $1000<M_{eff}$ (large MSAs). We note that the splitting up of the two datasets into those three groups are very different, with approximately equal sized subgroups for PSICOV, with 56, 47, and 47 as small, medium, and large MSAs, respectively, and skewed distributions for NOUMENON, with 97, 14, and 39 as small, medium, and large MSAs, respectively, i.e., with many more small MSAs. Results of the analyses of the importance of the parameter λ on all three groups of MSAs, for the two datasets we consider are shown in the same Figure 2. We observe the same behavior over all groups of MSAs, namely that the performance of the Gaussian model is improved as λ is increased, with a peak at λ=0.8. These results do mirror those presented in [27] that were computed using the same method, but on a different dataset of protein families. The overall performances however differ in the different groups, with a significant improvement in the large MSAs group compared to the small MSAs group, as intuitively expected. The same behavior is observed on the two datasets, however with much larger differences between the three groups of MSAs for the NOUMENON dataset. Results for the large MSA are very similar over the two datasets, indicating that a large information content in a MSA is usually sufficient to generate good contact prediction results, independent of additional structural information.

**Figure 3:**
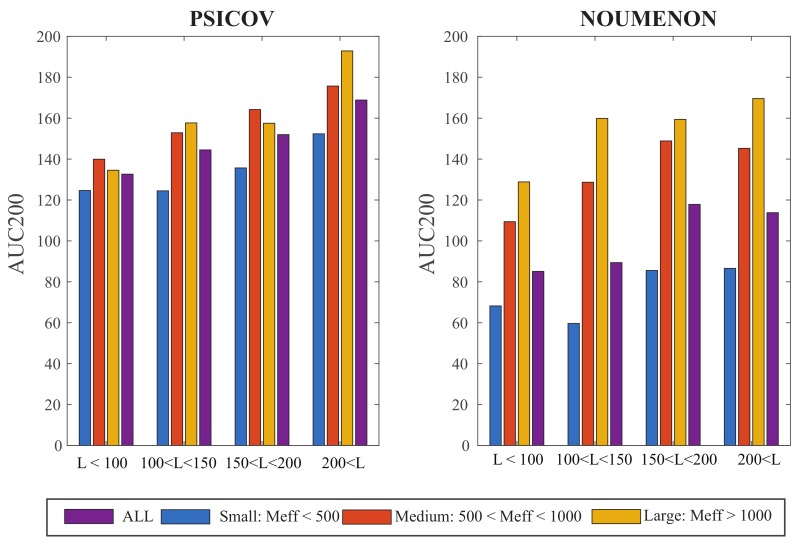
AUC200 plotted against the lengths of the multiple sequence alignments, for different groups of sizes of those alignments, for the PSICOV dataset (**left**) and the NOUMENON dataset (**right**). All computations are done with Gauss20, with λ=0.8. Results are shown over all three groups of MSAs with respect to size (see legend of Figure 2 for their definitions), as well as over four groups of MSA lengths (*x*-axis).

The MSAs in our dataset are characterized by two parameters, namely their size, *N* (or more appropriately their effective size, $M_{eff}$), and their length, *L*. We have seen above that the larger $M_{eff}$, the better the performance of GaussCovar. To assess the importance of *L*, we compared the averaged AUC200 values for GaussCovar over different ranges of *L*, over the three groups of MSA effective sizes defined above, and over the two datasets PSICOV and NOUMENON, with λ set to 0.8. Results are shown in Figure 3. The performance of GaussCovar improves as the lengths of the alignments increases, for the two datasets. This effect is more important for large MSAs, especially for the PSICOV dataset for which performances increase from an average AUC200 of 138 for short sequences, to an average AUC200 of 186 for long sequences. One possible explanation is that large proteins have larger cores; as co-variations seem to be concentrated in those cores (see [4]), it may explain the improvement observed as sequence length increases. Note however that these are averaged behaviors over specific groups of MSA lengths. When we plot the AUC200 directly versus the sequence length *L*, we find linear correlations between those two variables of 0.4, 0.49, 0.43, and 0.39 for the PSICOV MSAs in the small, medium, and large groups, and for all MSAs, respectively, and of 0.22, 0.54, 0.44, and 0.26 for the NOUMENON MSAs in the small, medium, and large groups, and for all MSAs, respectively. Those correlation coefficients are not significant.

As Gauss20 with λ=0.8 performs best on the two datasets of 150 protein families considered, it will serve as a reference in the following.

### 3.2. Amino Acid Representation 2: One Dimensional Property-Based Vectors

The 20D representation of amino acids described above only accounts for their alphabetic symbols and does not consider their physico-chemical properties. An alternate representation is to consider directly one such property. With this representation in mind, we have considered AAindex, a database of numerical indices representing various physicochemical and biochemical properties of amino acids [40,41]. The section AAindex1 of this database contains 566 different options for representing each amino acid as one numerical value, with each option corresponding to one property of amino acids. Out of those 566 indices, the 402 first originally assembled by Tomii and Kanehisa [45] have been clustered into six groups using single-linkage hierarchical cluster analysis. Those six groups, which we will refer to as clusters A, B, C, H, P, and O, mostly map with structural or physical properties of the amino acids: cluster A relates to alpha helix and turn propensities (118 indices), cluster B to beta sheet propensities (37 indices), cluster C to amino acid composition (24 indices), cluster H to hydrophobicity (149 indices), cluster P to physicochemical properties of the amino acids (46 members), and cluster O to other properties, such as the frequency of left-handed helices (28 members). The whole AAindex1 database, including the partitioning of the indices into these 6 clusters, is available at https://www.genome.jp/aaindex/.

**Figure 4:**
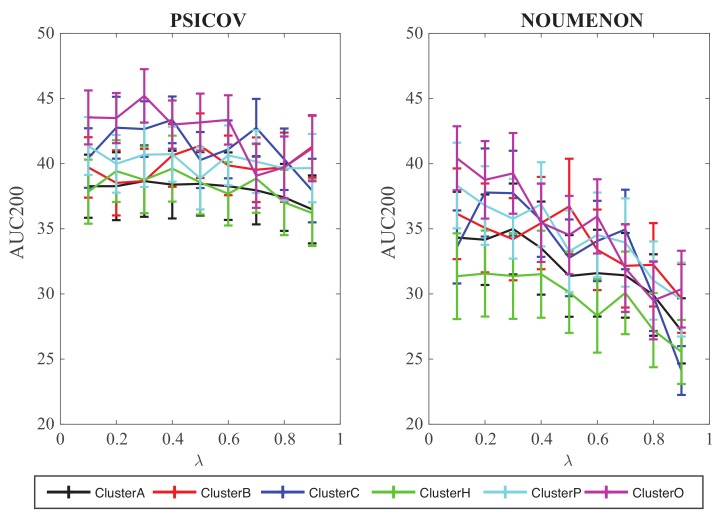
Performance of GaussCovar with 1D representations of amino acids over the PSICOV dataset (**left**) and the NOUMENON dataset (**right**). The predictive power of GaussCovar was tested using one of 402 amino acid properties available in the database AAindex [40,41]. Results are presented as averages over 6 groups of AAIndex1 scales, as originally defined in [45]. The 6 clusters A, B, C, H, P, and O correspond to indices related to alpha helix and turn propensities, beta sheet propensities, amino acid composition, hydrophobicity, physicochemical properties, and other properties, respectively.

We have analyzed the performance of GaussCovar with such a 1D representation of the amino acids over the two datasets PSICOV and NOUMENON, testing each of the 402 properties of AAindex1 for which cluster information is available separately. Each of those tests use a 1D encoding and is referred to as Gauss1D. Results are shown in Figure 4.

For each amino acid index, we explored the whole range of λ values between 0 and 1, with a step size of 0.1. The corresponding 402 curves of AUC200 as a function of λ were then regrouped based on the cluster id of the index, and summarized for each cluster by simple averaging. The conclusions from those experiments are three folds. First, the performance of GaussCovar with such a 1D representation is poor, with AUC200 around 40 for the PSICOV dataset, and around 35 for the NOUMENON dataset (those numbers should be compared to AUC200 values around 140 and around 100 for GaussCovar with the 20D amino acid representation, averaged over the PSICOV and NOUMENON datasets, respectively). GaussCovar with a 1D encoding still performs best on PSICOV, as observed for the 20D encoding. Clearly, a 1D representation decreases the differences between the amino acids, thereby reducing the performance of the method. Second, Gauss1D seems relatively insensitive to the choice of the weight of the prior λ when tested over the PSICOV dataset, while showing a clear trend of decrease in performance when the weight of the prior is increased for the tests on the NOUMENON datasets. Finally, it seems that the indices from cluster O, i.e., those based on “other” properties of amino acids, perform best both for the PSICOV and the NOUMENON datasets. It is unclear why this should be the case. We do note that the differences with the other clusters are small.

### 3.3. Amino Acid Representation 3: K Dimensional BLOSUM-Based Vectors

Not all contacts in proteins are specific. While hydrogen bonds are formed between residues within secondary structures, there are many non-specific hydrophobic contacts in the core of a protein, between those secondary structures. Hydrophobic contacts with an isoleucine, or with a leucine residue, for example, are very similar to each other. It is therefore natural to account for such similarities when identifying co-variations for residues in cores of proteins. Similarities between amino acids are usually derived from reference multiple sequence alignments and available under the format of a substitution matrix. Such a matrix stores the odds that any given amino acid can be replaced by any other. The BLOSUM matrices are among the most popular of those matrices [46,47]. They have been derived from reference BLOCKs sequence alignments, using different cutoffs in the sequence identity within a BLOCK. For example, the BLOSUM62 matrix is derived from alignments with sequences that are at most 62% identical; this matrix is considered to lead to good performance for database search [46].

**Figure 5:**
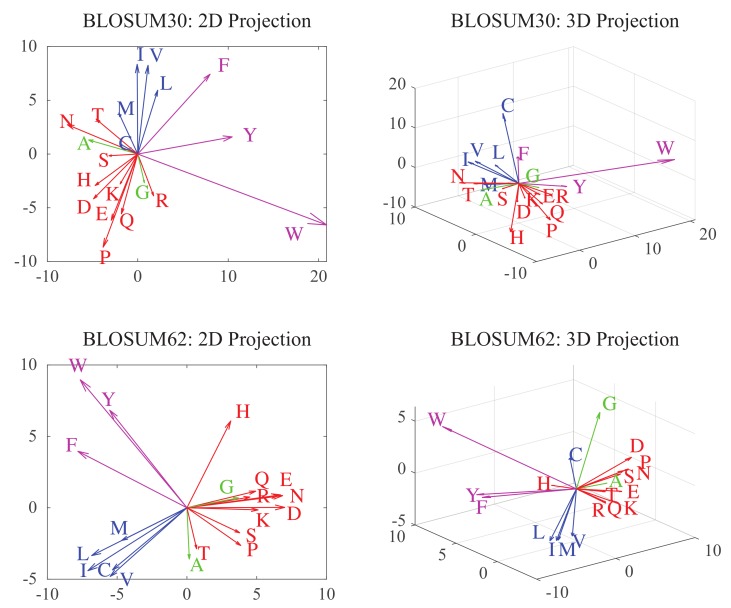
These plots represent the 2D (left panels) and 3D (right panels) vector representations of amino acids as derived from the BLOSUM30 (**top**), and BLOSUM62 (**bottom**) matrices. The proximity of these vectors relate to the chemical similarities of the amino acids they represent. To highlight this fact, we show the known polar amino acids (Q, R, E, K, H, N, D, T, P, and S) in red, the hydrophobic amino acids (M, V, L, I and C) in blue, and the aromatic amino acids (Y, F, and W) in magenta. Note that the two small amino acids, A and G (in green), stand out.

Substitution matrices describe each amino acid with a set of twenty numerical values, henceforth defining a twenty-dimensional space. Swanson was the first to embed this space into a plane, using a principal component analysis (PCA) approach. Following others (see [37] and references therein), we expand this concept and consider projections of BLOSUM substitution matrices on different spaces with varying dimensions. In the space of dimension *k* for example, amino acids are assigned *k* “coordinates” along the *k* principal components of the BLOSUM matrix considered. In Figure 5, we show the corresponding vectors in two and three dimensions for BLOSUM30 and BLOSUM62. For both matrices, the 3D representations of hydrophobic (in blue) and hydrophilic (in red) amino acids are well separated. There is however more overlap in the projections of the BLOSUM30 matrix between hydrophobic and aromatic amino acids (in magenta), and between hydrophilic amino acids and the two small amino acids A and G (in green).

**Figure 6:**
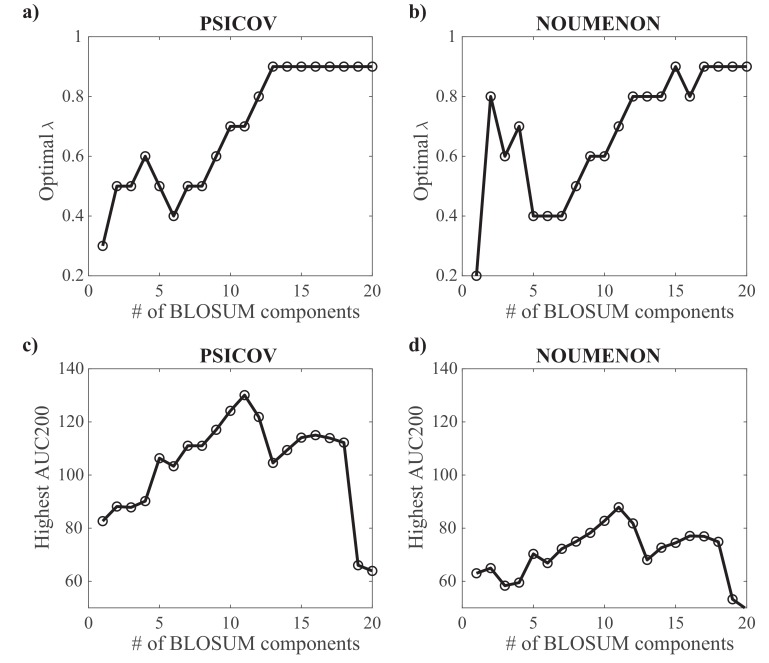
Parameterizing GaussCovar using a BLOSUM62-based encoding of amino acids. (**a**,**b**) The value of λ, the parameter controlling the amount of uniform prior included in the Gaussian model (see Equation (7)) that leads to optimal performance is plotted against the number of components for the vector representation of amino acids derived from the BLOSUM62 matrix, based on the PSICOV dataset (**a**), and based on the NOUMENON dataset (**b**). (**c**,**d**) The optimal AUC200 (over all predicted contacts) is given against the same number of components for the PSICOV dataset (**c**) and the NOUMENON dataset (**d**).

**Figure 7:**
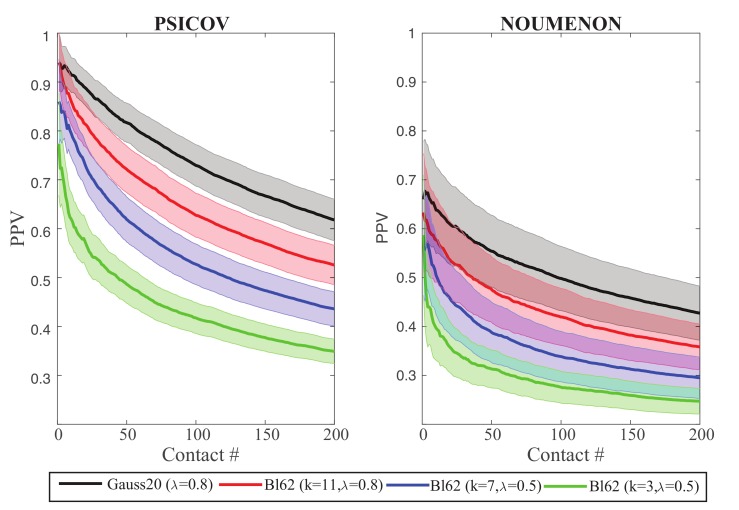
Precision Positive Values (PPV) plotted against the number of predicted contacts or the PSICOV dataset (**left**) and the NOUMENON dataset (**right**). Data were generated with GaussCovar, with amino acids represented with coordinates derived from the *k* principal components of the BLOSUM62 matrix. We show results for k=3,λ=0.5 (green), k=5,λ=0.5 (blue), and k=11,λ=0.7 (red). Data for GaussCovar with the 20D binarized encoding are shown in black for comparison. All PPV curves are arithmetic means over 150 protein families (see caption of Figure 1 for details). Shaded areas represent standard deviations.

We have analyzed the performance of GaussCovar using the BLOSUM62-based encoding of amino acids over the two datasets PSICOV and NOUMENON. We refer to this version of GaussCovar as BLO62. Results are shown in Figure 6 and Figure 7.

For each projection of BLOSUM62 onto spaces whose dimensions vary from 1 to 20, we have explored the whole range of λ values between 0.1 and 0.9, with a step size of 0.1. The corresponding $\lambda_{best}$ are shown in Figure 6a,b for the PSICOV and NOUMENON datasets, respectively. Not too surprisingly, the optimal λ value increases as the number of components *k* increases: large values of *k* lead to large covariance matrices for which the effects of under sampling are more important. Interestingly, while the performance of GaussCovar increases as the number of principal components *k* of the BLOSUM matrix considered increases between 2 and 11, the best performance is obtained for k=11, and not for larger *k* values, as observed on Figure 6b,d for the PSICOV and NOUMENON datasets, respectively. We note that larger values of *k* may have required larger values of λ (we stopped at 0.9). We did not explore this option, as it is unclear whether larger contributions of the prior in the Gaussian model are meaningful.

In Figure 7, we compare the PPV plots for GaussCovar based on BLOSUM62 vector representations of size k=3 (green), k=7 (blue), and k=11 (red), to the reference of using the binarized 20D representation of amino acids (black), over the two datasets PSICOV and NOUMENON. As expected from Figure 6, the BLOSUM-derived PPV plots get closer to the reference as the number of components increases, but never show improvements compared to this reference. As already observed for the Gauss20 and Gauss1D versions of GaussCovar, BLO62 performs better on PSICOV than on NOUMENON, highlighting again the possibilities of biases in the former.

**Figure 8:**
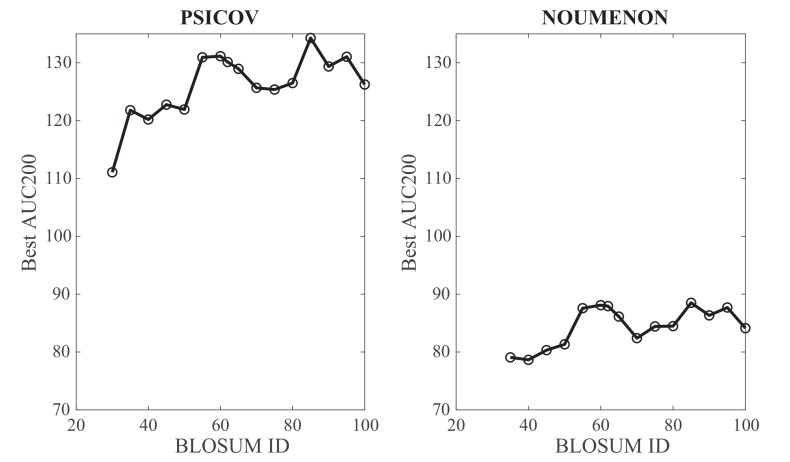
Influence of the BLOSUM matrix ID on the performance of GaussCovar based on BLOSUM encodings of amino acids. The optimal AUC200 (over all predicted contacts) for GaussCovar is plotted against the BLOSUM ID for the PSICOV dataset (**left**) and the NOUMENON dataset (**right**).

The BLOSUM62 matrix, intermediate between a more permissive matrix such as BLOSUM30 (computed over BLOCKS with a low sequence identity of 30%), and a restrictive matrix such as BLOSUM90 (from BLOCKS with up to 90% sequence identity) has become a standard in protein database searches and sequence alignments (see for example references [46,48] in which the performances of multiple substitution matrices were assessed). It was therefore natural to consider it for testing GaussCovar; it is unclear however if it would lead to the best results compared to other substitution matrices. We have repeated the analyses described above for BLOSUM matrices with IDs between 30 and 100. Note that BLOSUM100 is not the identity matrix: It is computed from BLOCKS with sequence similarities up to 100%. It is used however to detect sequences with very high similarities. The corresponding best performances of GaussCovar, as measured by AUC200 computed over all contacts from the mean PPV curves over all 150 protein families for the PSICOV and NOUMENON datasets, are plotted against the BLOSUM ID in Figure 8. Overall, it is observed that performance increases as the BLOSUM ID increases, for both datasets. Their results are consistent with the fact that the 20D binarized encoding performs best, as this encoding can be seen as derived from the identity matrix, i.e., a BLOSUM like matrix that focuses on perfect match, thereby closer to BLOSUM matrices with high IDs.

## 4. Discussion

In this work we have tested multiple numerical encodings of amino acids within the framework of contact predictions in proteins from a multivariate Gaussian analysis of multiple sequence alignments. We have shown that 1D encodings based on amino acid properties (from the AAindex databases), as well as higher dimension encodings computed from the principal components of BLOSUM substitution matrices, do not perform as well as a simple 20-dimensional binarized encoding in which each amino acid in represented as a vector of zeros, except at the position corresponding to its type, where the encoding is 1. We have also highlighted the importance of the prior when constructing the Gaussian model of a multiple sequence alignment, and have shown that as the dimension of the vectors representing the amino acids increases, more importance should be given to this prior in the model, even if it is based on a simple uniform distribution. In the original derivation of the Gaussian model [27], it was suggested that more informative priors could improve the prediction power of the Gaussian model. Our results indicate that this is not the case, at least when the information is based on substitution matrices.

The Gaussian model considered shares one similarity with the Potts model usually considered for covariation analyses in that it implicitly defines an energy for a sequence that is a sum of pairwise interactions between its residues, where those interactions are directly proportional to the “states” of those residues, their encodings using the terminology considered in this work. It is unclear however if a direct proportionality is optimal. We are currently testing other formats for the energy of a sequence, still within the context of numerical encodings of amino acids.

Finally, we note that progress in geometric contact predictions in proteins based on sequence information only may not come solely from improvements of the models used to analyze MSAs. The multivariate Gaussian model considered here, just like the Potts Hamiltonian model [6,21] summarizes all forms of interactions between positions in the MSA into pairwise contacts. As the information content of an MSA goes beyond those simple interactions, it was natural to see attempts to combine such additional information to pairwise co-variation measures by using machine-learning techniques. It is interesting, for example, that out of the 23 methods that have been used for contact predictions in CASP12, at least 21 are clearly based on machine learning, using different versions of deep learning methods or combinations of such methods [9]. Those methods were reported to be strikingly successful, and precisions above 90% were achieved by the best predictors in more than half the targets in CASP12 [9], a result considered to be a highly significant improvement compared with the results obtained during CASP11 [49].

## 5. Materials and Methods

### 5.1. Vector Representations of Amino Acids

In addition to the common 20-dimensional binarized representation of amino acids (in which an amino acid of type *i* is represented with a vector of 20 zeros, except at position *i* where the vector component is 1), we have used two different vector representations of amino acids in this work, one based on their physical properties, and one based on substitution matrices.

In our first representation, each amino acid is encoded by one real index value, as defined in AAindex [40,41]. AAindex is a database of numerical indices representing various physicochemical and biochemical properties of amino acids and pairs of amino acids. We have used the former, and have tested the 402 properties that were available in 1996 [45]. Those are the first indices available in AAindex and accessible online at https://www.genome.jp/aaindex/. We have used those 402 values, and not all 566 currently available in AAindex, as the classifications of those 402 into six groups of properties is available. Those six properties are α and turn propensities (Group 1), β propensity (Group 2), composition (Group 3), hydrophobicity (Group 4), physico-chemical properties (Group 5), and other properties (Group 6), as defined in Ref. [45].

Common measures of similarities between amino acids are usually presented in the form of a substitution matrix, which stores the odds that any given amino acid can be replaced by any other. Substitution matrices can be compiled based on substitutions observed in protein sequence families (for review, see [47], or directly from amino acids physico-chemical properties (see, for example, [45]). Substitution matrices describe each amino acid with a set of twenty real values, thereby defining a twenty-dimensional space. Swanson was the first to embed the space corresponding to the original PAM matrix [50] into a plane, using a principal component analysis (PCA) approach [29]. Since then, different embedding of the BLOSUM62 matrix [46] into feature spaces of reduced dimensions have been proposed, usually noticing that three dimensions already produce a reasonably good approximation of the high dimensional amino acid space (see [37] and reference therein). We have used the same scheme to generate embeddings of different BLOSUM matrices, with different dimensions, using the same PCA strategy applied in the studies mentioned above.

### 5.2. GaussCovar: Residue Contact Predictions from a MSA

GaussCovar, our implementation of the Gaussian modeling of multiple sequence alignment mirrors the algorithm described in [27]. For sake of completeness, we provide an outline of this algorithm, briefly describe its main steps, and highlight the differences with the published method.

Step 1b is required to remove (to some extent) the dependencies between the sequences in the MSA. We use the same re-weighting scheme as the one used in PSICOV [26], which was inspired by similar re-weighting schemes proposed in Refs. [21,23]. The procedure is fully described in the Supplemental material of [27]. We only provide basic details here, as they are needed to clarify the concept of effective number of sequences in a MSA, $M_{eff}$, used in the Results section. To generate the weights, we first compute a similarity cutoff *r*. This cutoff is defined as being inversely proportional to the average sequence identity over all pairs of sequences in the MSA. Once this cutoff *r* has been computed, it is used to define groups of similar sequences around each sequence: only sequences with less than rL identical amino-acids are considered to carry independent information. For each sequence $S_{n}$ in the MSA we count the number $m_{n}$ of sequences with at least rL identical amino-acids; the weight of the sequence is then defined as $w\left(n\right)=1/m_{n}$. The effective number $M_{eff}$ of sequences is the sum of the weights over all sequences:

(8) $$M_{eff}=\sum_{n=1}^{N}w\left(n\right)$$

The main difference between our algorithm and the original algorithm described in [27] is step 2, in which we allow for a broader range of possible encodings of amino acids into vectors. As described above, we have considered encoding an amino acid as a single real value, as defined in one of the scales available in the AAindex database [40,41], projected version of BLOSUM matrices onto spaces of varying dimensions, as well as the original binarized representation used by Baldassi et al.

In step 3, the weighted empirical mean and covariance over the MSA are computed as:

(9) $$\bar{X}=\frac{\sum_{n=1}^{N}w_{n}X_{n}}{\sum_{n=1}^{N}w_{n}}$$

and

(10) $$\bar{C}=\frac{1}{\sum_{n=1}^{N}w_{n}}\sum_{n=1}^{N}w_{n}{\left(X_{n}-\bar{X}\right)}^{T}\left(X_{n}-\bar{X}\right)$$

The score P(i,j) between two positions *i* and *j* is computed from the Frobenius norm of the submatrix J(i,j) of side s×s, where *s* is the size of the vector representing amino acids. (step 6 of the algorithm). The Frobenius norm is not independent of the gauge choice (see [25] for a full discussion of this point). We apply the following two steps to compute $P_{ij}$:

(11) $$\begin{array}{ccc} J_{ij}^{c}\left(k,l\right) & = & J_{ij}\left(k,l\right)-\left(J_{ij}1_{s}\right)\left(k\right)-\left(J_{ij}^{T}1_{s}\right)\left(l\right)+\left(1_{s}^{T}J_{ij}1_{s}\right)\\ P_{ij} & = & \sqrt{tr(J_{ij}^{c}{\left(J_{ij}^{c}\right)}^{T})} \end{array}$$

where $1_{s}$ is a vector of *s* ones.

Step 7 applies an average-product correction (APC) to the score matrix, initially introduced in [39] to supper effects from phylogenetic biases, and introduced by Jones et al. for covariation analyses [26]. The APC-corrected score is computed as:

(12) $$P^{APC}=P-\frac{\left(P1_{L}\right){\left(P^{T}1_{L}\right)}^{T}}{1_{L}^{T}P1_{L}}$$

where $1_{L}$ is a vector of *L* ones.

**Algorithm 1** GaussCovar: extracting residue contacts from a multiple sequence alignment.**Input:** a MSA with *N* sequences, of length *L*, *t*, the type of amino acid encoding, and λ, the weight of the prior;
(1)Preprocess MSA:(a)Cleanup: remove sequences with more than 90% of gaps(b)Assign weight w(n) to each sequence $S_{n}$ in the MSA(2)Convert each letter-based sequence $S_{n}$ of length *L* into a real-valued vector $X_{n}$ of length Ls, as defined by the encoding scheme *t*
(3)Compute empirical mean $\bar{X}$ and covariance matrix $\bar{C}$ using weighted versions of Equations (1) and (2)(4)Compute corrected covariance matrix $C_{M}$ by adding a prior, using Equation (7). The weight of the prior is controlled by λ
(5)Compute $J=C_{M}^{-1}$
(6)For each pair of positions *i* and *j* in the MSA, compute a score $P_{ij}$ as the Frobenius norm of the submatrix $J_{ij}$ of size s×s.(7)Apply APC correction to the score matrix *P*
(8)Rank all pairs (i,j) with j−i>4 in decreasing order of corrected scores $P_{ij}^{APC}$. The top 200 pairs defined the predicted contacts.


### 5.3. Assessing the Performance of GaussCovar

In order to assess the correctness of the contacts predicted by GaussCovar, we need in addition to the MSA a “gold standard”, or reference protein structure that best represents the proteins in this MSA. A contact predicted by GaussCovar between two positions *i* and *j* is deemed correct if the two corresponding residues *k* and *l* in the reference protein structure are distant by less than 8 Å, where the inter-residue distance is defined as the minimum distance between any heavy atoms of the two residues. Note that this corresponds to the definition of success proposed by Morcos et al. [21]. Other definition of success are possible (see for example [26]). Each predicted contact is then assigned as true or false positives based on this criterium.

The precision or positive predictive value (PPV) of GaussCovar on a given MSA is a discrete function determined by computing PPV(k) = (true positives)/(true positives + false positives) where *k* is the number of predicted contacts. A perfect prediction would generate a curve such that PPV(k)=1 for all *k*, while a poor prediction would see PP(k) close to zero for all *k*. We quantify the overall quality of the prediction as the area below this curve, which we write AUC200, as it is computed over the first 200 predicted contacts. We note that the larger the AUC200 score, the better the prediction. The AUC200 score for a perfect prediction is 200.

To assess the significance of the performances of GaussCovar with different amino acid encodings over a dataset, we generated a mean curve PPV, averaged over all families included in the MSA. At each contact level *k*, we computed a mean value and a standard deviation. To compare two such curves (say when comparing the performance of GaussCovar under certain conditions with the performance of the program PSICOV), we applied a two tailed Student’s t-test at each level of contact *k*. The two underlying distributions contain the same number of samples (i.e., 150 families). The difference between the two curves at a given *k* value is assumed to be statistically significant if the corresponding *p*-value is smaller than 0.05. if all *p*-values over all levels *k* are smaller than 0.05, the two curves themselves are considered statistically different.

### 5.4. Test Datasets

We have used two test sets for assessing the predictive abilities of GaussCovar under different representations of amino acids, namely the original PSICOV dataset [26] and the recent NOUMENON dataset [42].

The PSICOV set is a carefully designed dataset composed of 150 protein families, originally generated as a test set for the namesake program PSICOV (Protein Sparse Inverse COVariance). It was designed as follows (for full details, see [26]). One hundred and fifty proteins whose structures are available in the Protein Data Bank (PDB, http://www.pdb.org) [2] and lengths between 75 residues and 275 residues were selected. All the structures of those proteins were of high resolution (≤ 1.9 Å) and all are known to be biological monomers, to eliminate the risks of detecting inter-chain contacts. The sequences of those proteins are dissimilar. For any pair of sequences, a sequence alignment using the SSEARCH package from the FASTA package (https://fasta.bioch.virginia.edu/) has a E-value greater than 10${}^{-5}$, and usually greater than 0.1. For each sequence, multiple sequence alignments were generated using the jackhammer program from the HMMER 3.0 package (http:hmmer.org). The sequence was checked against, and aligned with, homologues from the UNIREF100 data bank, using three iterations of jackhammer. The resulting alignments contain between 511 and 74836 sequences. Those alignments, as well as the corresponding reference PDB structures, and the results of running PSICOV on them, are available at http://bioinfadmin.cs.ucl.ac.uk/downloads/PSICOV/ as Supplementary Materials of the original PSICOV paper [26].

The NOUMENON set is a more recent dataset that was designed to provide a bias-free test set for assessing the performance of residue contact prediction methods [42]. It also contains 150 proteins and was derived in a similar manner that PSICOV was built. Namely, 150 sequences of proteins with known highly resolved structures (≤2 Å) were selected. The lengths of these proteins also vary between 75 residues and 275 residues. It was also ensured that their structures have at least *N* contacts, where *N* is the length of the protein. Each of those sequences was then checked against, and aligned with, homologues from the UNIREF100 data bank, using three iterations of jackhammer. The key difference with the PSICOV dataset is how the initial 150 proteins are selected. This selection is constrained such that their homologues have no, or little evolutionary relationship with proteins whose structures are available in the PDB. The NOUMENON dataset can be retrieved at http://ibsquare.be/noumenon.

## Abbreviations

The following abbreviations are used in this manuscript:

MSA	Multiple Sequence Alignment
PCA	Principal Component Analysis
